# Importance of Argan Oil in Human Health According to the Dosage of Antioxidants in the Algerian Argan Fruits (*Argania spinosa)*

**Published:** 2020

**Authors:** Zohra Benaouf, Imen Benbahi, Oussama Djorf, Zahira Souidi, Reda Kechairi

**Affiliations:** 1. Research Laboratory in Geo Environment and Spatial Development LGEDE, University of Mustapha Stambouli, Mascara, Algeria; 2. Laboratory of Plant Ecology and Environment, Faculty of Biological Sciences, USTHB University, Bab Zouar, Algiers, Algeria; 3. Laboratory of Biochemistry, Faculty of Chemistry, USTHB University, Bab Zouar, Algiers, Algeria; 4. Research Laboratory on Biological Systems and Geomancy (L.R.S.B.G), University of Mustapha Stambouli, Mascara, Algeria; 5. Laboratory of Plant Ecology and Environment, Faculty of Biological Sciences, Telemcen University, Telemcen, Algeria

**Keywords:** Antioxidants, Argan oil, Health, Human, Morocco

**Figure 1.**
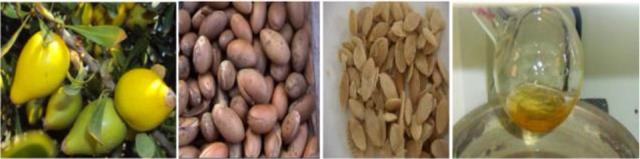
Fruit, seed, core of argan oil.

The chemistry and a few pharmacological aspects of argan oil have been studied; there are still no strong clinical data available that provide evidence of the efficacy of argan oil in humans. That argan oil constituents have pharmacological properties *in vitro* is not sufficient to ascertain the clinical potential of whole argan oil (Figure 1). More studies are necessary to determine its impact on human health. In Tindouf (Algerian area) and Morocco, the position of argan oil as a natural product with strong consumer expectations resulting from traditional claims of activity that are insufficiently supported by scientific proof is shared by several other plant extracts or products. Such a trend is likely to continue in view of the strong current demand for food supplements. This demand justifies pharmacological studies on these products 1,2. Argan oil has a high level of oleic and linoleic acids and antioxidant compounds which has impact on cardiovascular diseases 3. Minor compounds of argan oil, such as sterols, may be involved in its cholesterol-lowering effect 4. The antidiabetic effect of argan oil has been claimed for a long time in traditional medicine; however, the mechanism of regulation of the level of glucose in the blood remains unknown 5. The antihypertensive effect of argan oil and its mechanism of action have been studied by Berrougui *et al* 6.

Our results show that the phenolic fractions studied have remarkable antioxidant properties. Although the composition of the phenolic fraction of fruits can evolve over the years, they deserve a better valuation in the pharmacological, cosmetic and agro-food fields because of their antioxidant properties.
